# Insight into the Progress on Natural Dyes: Sources, Structural Features, Health Effects, Challenges, and Potential

**DOI:** 10.3390/molecules27103291

**Published:** 2022-05-20

**Authors:** Nannan Li, Qirou Wang, Jingna Zhou, Shuqin Li, Junyu Liu, Haixia Chen

**Affiliations:** Tianjin Key Laboratory for Modern Drug Delivery & High-Efficiency, School of Pharmaceutical Science and Technology, Tianjin University, Tianjin 300072, China; 13234039725@163.com (N.L.); wangqirou110@163.com (Q.W.); jingnazhou66@163.com (J.Z.); lisq2013@sina.com (S.L.); junyuliu@tju.edu.cn (J.L.)

**Keywords:** natural dyes, structure features, pharmacological activities, development strategies

## Abstract

(1) Background: Dyes play an important role in food, medicine, textile, and other industries, which make human life more colorful. With the increasing demand for food safety, the development of natural dyes becomes more and more attractive. (2) Methods: The literature was searched using the electronic databases PubMed, Web of Science, and SciFinder and this scoping review was carried out following Preferred Reporting Items for Systematic Reviews and Meta-Analyses (PRISMA). (3) Results: 248 articles were included in this review. This review summarizes the research progress on natural dyes in the last ten years. According to structural features, natural dyes mainly include carotenoids, polyphenols, porphyrins, and alkaloids, and some of the newest dyes are summarized. Some pharmacological activities of carotenoids, anthocyanin, curcumin, and betalains in the last 10 years are summarized, and the biological effects of dyes regarding illumination conditions. The disadvantages of natural dyes, including sources, cost, stability, and poor bioavailability, limit their application. Here, some feasible strategies (potential resources, biotechnology, new extraction and separation strategies, strategies for improving stability) are described, which will contribute to the development and utilization of natural dyes. (4) Conclusion: Natural dyes show health benefits and potential in food additives. However, it is necessary for natural dyes to pass toxicity tests and quality tests and receive many regulatory approvals before their final entry into the market as food colorants or as drugs.

## 1. Introduction

Dyes play an important role in food, medicine, textile, and other industries, which make human life more colorful. Dyes are divided into natural and synthetic dyes according to their source. However, many synthetic colorants have environmental toxicity and threaten human health. These adverse effects of synthetic colors have made the scientific community skewed toward natural colors [1]. With the increase in the demand for natural dyes in food, cosmetics, and other fields, it is of important value to develop natural dyes, especially in the food field.

Natural dyes are widely found on land and in the sea, and can be extracted from plants, animals, microorganisms, minerals, and some other materials. Most mineral dyes cannot be used in the food industry because they are harmful to humans. Most plant dyes, animal dyes, and microbial dyes are not only safe and reliable, but also have functions of nutrition and pharmacological activities such as antioxidant, anti-inflammatory, anti-cancer, anti-obesity, anti-microbial, and anti-viral effects. The use of natural dyes has a long history, for example, indigo, which is extracted from plants, has been used for thousands of years [2]. In addition to being classified by source and color [3], natural colorants are divided into the major categories by chemical structure, such as indole derivatives (quinones and violacein), alkaloids, polyenes, macrolides, peptides, or terpenoids [4]. In recent years, some new natural dyes have been isolated, and new activities, mechanisms, and new applications have been explored.

This review summarizes the research progress on natural dyes in the last ten years. Information in the last 10 years (from 2012 to 2022) was searched using the databases PubMed and Web of Science, structures of compounds were checked with the database SciFinder, and the review was carried out according to the Preferred Reporting Items for Systematic Reviews and Meta-Analyses (PRISMA) guidelines [5,6]. The category of natural dyes and main compounds are listed according to the literature, and different categories of dyes were used as terms to retrieve related bioactivities (Table 1). This review involves some new natural dyes and development strategies of natural dyes, which provide insight for further development and potential applications of the natural dyes.

## 2. Results

### 2.1. Literature Search Results

**Figure 1:**
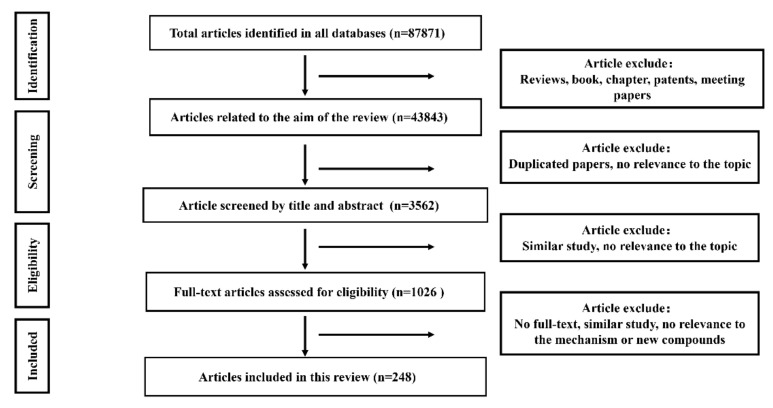
The flowchart of the selection process of literature based on PRISMA.

The flowchart of the literature search and selection of this review is shown in Figure 1. Overall, 87,871 studies were identified. Then, 44,028 records were removed for the following reasons: reviews, book chapters, letters, news, patents, meeting papers, reports, etc. Duplicated papers and records that are not relevant to the topic were excluded after the database screening, and 3562 studies were identified. By screening the titles and abstracts, 2536 records were excluded because they were similar and had no relevance to the scope of this review. Then, the full text of 1026 papers was reviewed and assessed to check if they were eligible for this scoping review. As a result, 248 studies were included in this review.

### 2.2. Resources

**Figure 2:**
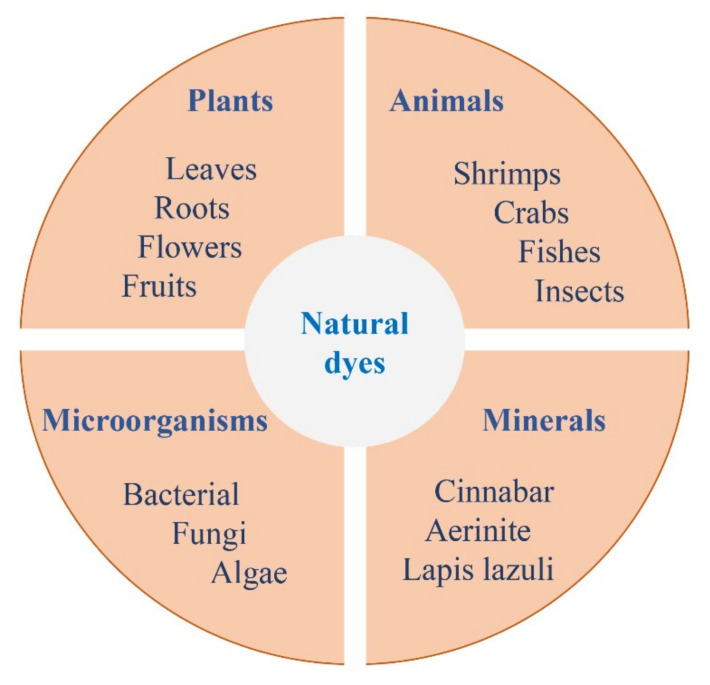
Resources of natural dyes including plants, animals, microorganisms, and minerals.

Natural dyes can be extracted from plants, animals, microorganisms, minerals, and some other materials (Figure 2). 

#### 2.2.1. Plants

In nature, the leaves, roots, flowers, and fruits of plants are all important sources of natural dyes [7,8,9] and these natural dyes determine the color of different parts of plants. For example, chlorophylls are responsible for the green color of leaves, carotenoids are responsible for yellow and red flowers, vegetables, and fruits, while anthocyanins determine the color of flowers and fruits from orange to dark blue [10]. The color of mangoes and tomatoes is associated with carotenes and lycopene, respectively [11,12]. Cornflowers, blueberries, mulberries, and strawberries are rich in anthocyanins. Betalains present in vacuoles of plants are responsible for beetroot’s deep red or yellow color [13]. Seasons change, plant leaves change from green to yellow or red, and the changes in leaves’ color during leaf senescence depend on the different combinations of chlorophylls, carotenoids, and anthocyanins [14,15]. Most of the sources of plant dyes are leaves, flowers, fruits, and roots, which are renewable resources and can be used as a source of natural dyes.

#### 2.2.2. Animals

Animals are also sources of natural dyes. The most common ones are carmine acid, astaxanthin, and lac dyes. Carmine acid, varying from pink to reddish-purple, is a natural dye extracted from the dried bodies of females of the insect species Dactylopius coccus Costa (cochineal). Carmine acid has been used in food, cosmetics, medicine. and textile production and is allowed by the food laws in different countries [16,17]. Astaxanthin is widely found in nature, especially in aquaculture products such as shrimp, crab, and fish [18]. Astaxanthin, as a red–orange ketocarotenoid, has excellent antioxidant activity [19] and it is widely used as a color additive in production [20]. Lac dyes are pink–red–purple organic colourants derived from an insect and contain several components, such as laccaic acid A, laccaic acid B, laccaic acid C, laccaic acid D, and laccaic acid E [21]. Lac dyes have been used for textiles and painting for thousands of years [22]. Recently, some new quinone dyes were isolated from the deep-sea crinoid Hypalocrinus naresianus [23,24]. With the development of marine resources, deep-sea animals are expected to become potential new sources of natural dyes.

#### 2.2.3. Microorganisms

Microorganism communities are the most widely distributed living organisms. They are closely connected with animals, plants, and other microorganisms in the form of saprophytic states, symbiosis, and parasitism, and constitute an important part of the biosphere and ecosystem [25]. Microorganisms, including bacteria, fungi, and some algae, are an important source of natural dyes [26,27]. At the moment, an extraordinary range of microbial pigments in various environments is available, such as carotenoids (β-carotene, canthaxanthin, astaxanthin), bacteriochlorophylls, flavins, indigoids, melanins, pheomelanin, prodigiosin, violacein, glaukothalin, phycocyanin, xanthomonadin [28,29,30]. Fungi have been identified as potential dye producers, and some pigment-producing fungi are as follows: *Aspergillus niger*, *Aspergillus versicolor*, *Monascus* sp., *Trichoderma viride, Penicillium purpurogenum* [31], *Aspergillus sydowii*, *Aspergillus aureolatus*, *Aspergillus keveii*, *Penicillium flavigenum*, *Penicillium chermesinum*, *Epicoccum nigrum*, *Lecanicillium aphanocladii*, and *Fusarium sp.* [32]. Some of the fungal dyes have already been used as food colorants in the market, such as Monascus pigments, arpink red from *Penicillium oxalicum*, riboflavin from Ashbyagossypii, and β-carotene from *Blakeslea trispora* [33,34]. Since the 1880s, Monascus pigment has been widely used as food coloring throughout the world [35]. In Asia, Monascus pigment has been widely utilized in food industries, especially in China and Japan [36]. Compared to plants and animals, fungi show immense advantages in production and cost. For example, fungal dyes are season-independent and can grow in a cheap culture medium easily and fast. In particular, fungal dyes show good stability, solution, different color shades, and easy processing [37]. Microbial dyes are expected to replace synthetic colorings and, since most of them are eco-friendly and nontoxic to humans, they can be used for application as food additives and in the medicinal field. Fungi could be a good and readily available source of natural dyes.

#### 2.2.4. Minerals

Mineral dyes are refined from natural minerals and are mainly used for painting, handicrafts, antiques, and restoration of cultural relics. Since ancient times, cinnabar (HgS) has been used as a red dye, widely used in the art of ancient Rome, for adornment, and in medieval manuscripts of colored drawing or patterns [38]. Aerinite is a light-blue mineral, which comes from local ores in the southern Pyrenees. Blue dye can be created from aerinite and was used in Romanesque wall paintings in Andorra and Catalonia, Spain [39]. Natural ultramarine (UMB) dyes from lapis lazuli have been used in the past [40]. The UMB dyes, as one group of mineral dyes, are characterized by sodalite structure and colored sulfur species as chromophores are encapsulated inside. The general formula of UMB dyes is Na_8_[AlSiO_4_]_6_[S_2_S_3_]_2_ [41]. Ancient painters, potters, and craftsmen obtained blue, green, red, and black colors from rocks or minerals. Therefore, minerals dyes are more suitable for use in art and architecture than food additives.

### 2.3. Structural Features of Natural Dyes

**Figure 3:**
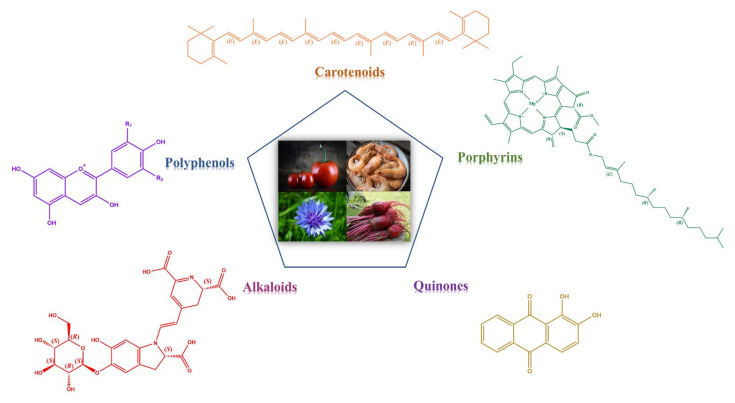
The major categories of natural dyes divided by chemical structure.

Natural dyes can be divided into the major categories by chemical structure. They mainly include carotenoids, polyphenols, porphyrins, alkaloids, and quinones (Figure 3).

#### 2.3.1. Carotenoids

**Figure 4:**
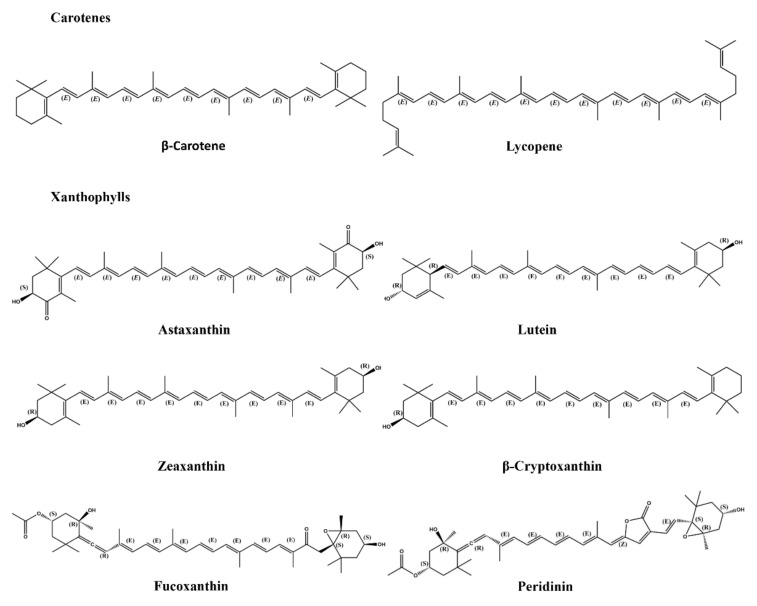
The structures of carotenoids including carotenes and xanthophylls.

Carotenoids are tetraterpene, liposoluble, and yellowish-orange dyes, and the difference in colors depends on the specific conjugated double-bond structure of molecules [42]. Carotenoids, as the most widely distributed dyes in nature, are found in microorganisms (photosynthetic bacteria, some species of archaea), plants (leaves, fruits, flowers), and animals (birds, insects, fish, and crustaceans) [8]. According to the length of their carbon backbone, carotenoids have been classified as C30, C40, and C50 carotenoids [43]. C40 carotenoids have eight isoprene molecules and the yellow, orange, and red hues depend on an extensively conjugated polyene chain. This characteristic chemical structure is responsible for their physiological function as an antioxidant and provitamin A nutrient, as well as their ability to protect from UV radiation [44]. Carotenoids are divided into two groups: carotenes and xanthophylls (Figure 4). Carotenes, such as α-carotene, β-carotene, γ-carotene, and lycopene, are hydrocarbons. About 50 kinds of carotenes have been found in nature [45]. β-carotene can be converted into vitamin A after entering the body, which is an important vitamin for humans and it can help prevent eye damage and protect skin [46]. Xanthophylls are carotenoids containing oxygen atoms, such as astaxanthin, lutein, zeaxanthin, β-cryptoxanthin, fucoxanthin, and peridinin. Structures of xanthophylls show marked diversity and about 800 kinds of xanthophylls have been reported in nature up until 2018 [8]. Those molecules of xanthophylls include hydroxy, carbonyl, aldehyde, carboxylic, epoxide, and furanoxide groups. They assist in photoprotection and light harvesting, and play potential roles in the photosynthetic system. In fact, some xanthophylls have been widely used in various fields. For example, natural astaxanthin has been approved as a food colorant in fish and animal feed by the FDA [47]. Canthaxanthin is also widely applied in food, cosmeceutical, pharmaceutical, fishery, poultry, and other industries [48]. Canthaxanthin as a food additive in fisheries could improve the color of shrimp and salmonid fish [49]. In addition, zeaxanthin and lutein are considered to play a potential role in maintaining eye health [50]. In recent years, several carotenoids have been isolated (Table 2), but more attention has been paid to screening strains from nature that can produce carotenoids. Carotenoids are widely produced by microorganisms and plants. Carotenoids as essential components of the human body have great development value. The health benefits of carotenoids have also been widely studied.

#### 2.3.2. Polyphenols

Anthocyanin is one of the most representative polyphenol natural dyes. Anthocyanins and their glycosides, as species of water-soluble pigments, are ubiquitous in the plant world. More than 250 kinds of anthocyanin molecules have been found from agricultural products and they are responsible for the colors of products such as blueberry, black wolfberry, cherry, black raspberry, strawberry, grape, and purple and red corn [80]. The color of anthocyanin depends on the pH of the solution, which might be due to the transformation of anthocyanin structure in different pH conditions [81]. For example, roselle anthocyanins became red at pH 2.0–3.0 because the anthocyanins mainly were present in the form of yellow salt ions; the red decreased at pH 4.6–6.0 with the structures transforming into blue quinonoidal base; at pH 7.0–9.0 the structures transformed to colorless pseudo-base and the color gradually turned to blue. When the pH value was greater than 9.0, anthocyanins turned to yellow-green due to the degradation in the strongly alkaline conditions [9,82]. Based on this characteristic, anthocyanins can be developed as indicators to monitor the freshness of food and have great potential in intelligent packaging.

**Figure 5:**
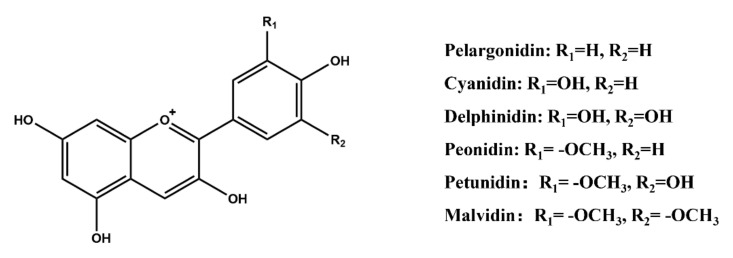
Molecular structures of the main anthocyanins.

Pelargonidin, cyanidin, delphinidin, peonidin, petunidin, and malvidin are the six main anthocyanin compounds (Figure 5), and they differ in the number and positions of the substituents in the benzene ring [83]. However, anthocyanins are present in nature in the form of glycosides because free anthocyanins are unstable. Anthocyanins often form 3-glycoside or 3,5-diglycoside compounds with one or more glucose (the most common), rhamnose, galactose, xylose, arabinose, etc. through glycosidic bonds [84,85]. The application of anthocyanins in foodstuffs has been approved in many countries, including in Europe (EU E No. E163), the United States, and Japan, and they are mainly used in beverages, confectionery, baking, frozen snacks, and dairy and fruit products [86]. Some new anthocyanins have been isolated and identified in recent years (Table 2), and anthocyanin synthesis-related genes in different fruits and plants have also been extensively studied, which provide a new strategy for anthocyanin development.

**Figure 6:**
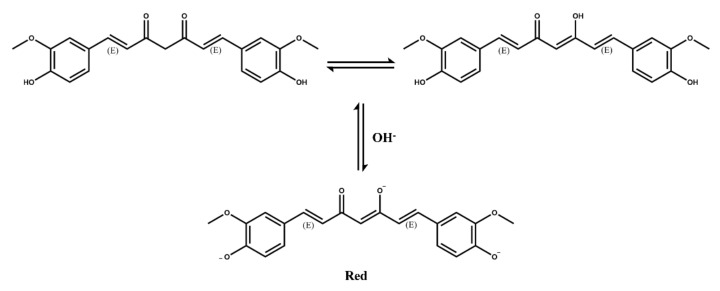
Molecular structures of curcumin and the color changes in acid–base conditions.

Curcumin is a polyphenolic compound extracted from Curcuma longa L., which has been generally recognized as safe [87]. Curcumin has also been approved by the FDA as a natural food additive. Curcumin can be used as an acid–base indicator because it changes from yellow to red at pH greater than 8 (Figure 6). Anthocyanin and curcumin are the main phenolic natural dyes and promising natural dyes because they have been shown to have many health benefits. Curcumin also plays a potential role in intelligent packaging [88].

#### 2.3.3. Porphyrins

**Figure 7:**
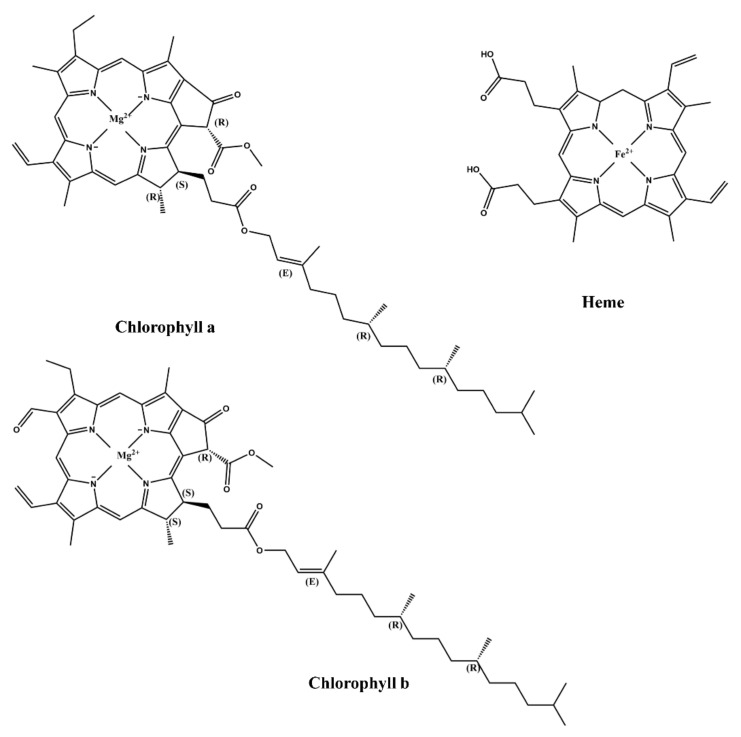
Molecular structures of heme, chlorophyll a, and chlorophyll b.

Porphyrin dyes are also known as tetrapyrrole derivatives and mainly include heme and chlorophylls (Figure 7). Chlorophylls are magnesium-tetrapyrrole molecules and the major photosynthetic greenish pigments found in algae, plants, and cyanobacteria that play essential roles in photosynthesis [89,90]. Chlorophylls mainly include five types: chlorophyll a, chlorophyll b, chlorophyll c, chlorophyll d, and chlorophyll f. Chlorophyll a and b play a dominant role in photosynthetic organisms, and chlorophyll a is essential in photochemistry. Chlorophyll a as a blue/green pigment with maximum absorbance from 660 to 665 nm and is the main pigment of phytoplankton; it is considered to indicate the rhythm of marine ecosystems [91]. Chlorophyll b is converted to chlorophyll a via 7-hydroxymethyl chlorophyll a [92]. Chlorophyll d is found in marine cyanobacteria and red algae, and chlorophyll f, found in various genera of cyanobacteria, co-occurs with chlorophyll a [90,93]. Chlorophyll d and chlorophyll f show red-shifted absorption features compared to chlorophyll a and chlorophyll b because of the position of formyl substitutions. The maximal absorptions of chlorophyll d and chlorophyll f are 697 nm and 707 nm in methanol, respectively [93]. Sodium copper chlorophyllin, a green colorant, has been approved by the USA FDA for use only in “citrus-based dry beverage mixes in an amount not exceeding 0.2 percent of the dry mix” (http://www.ecfr.gov/cgibin/textidx?SID=6ffd146f772f44d1b7b76af13be18518&node=21:1.0.1.1.27.1.31.12&rgn=div8 (accessed on 5 May 2020)).

#### 2.3.4. Alkaloids

**Figure 8:**
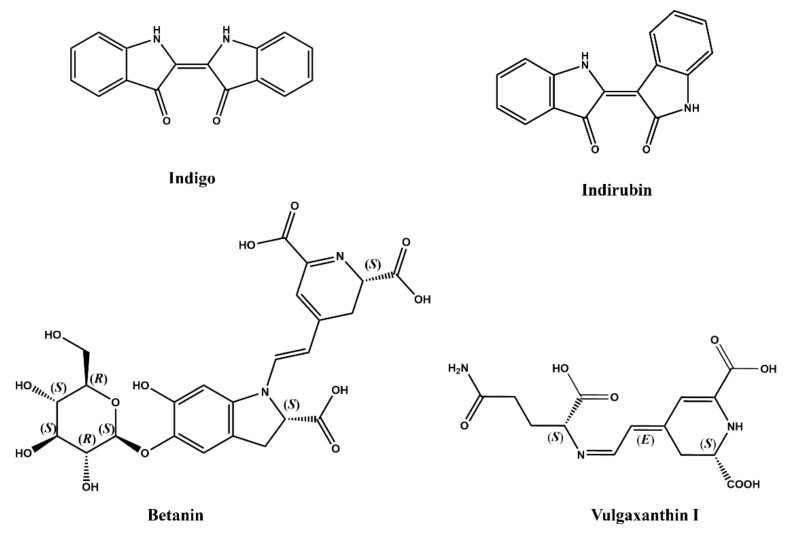
Structure of alkaloid pigments including indigo, indirubin, and betalains.

Betalains as water-soluble natural plant dyes have been recognized as red food-coloring agents due to no toxicity and health benefits [94]. Betalains contain several dye compounds and those compounds contain nitrogen. The dye compounds in betalains mainly include violet betalains such as betanin and yellow betaxanthins [95], and betanin and vulgaxanthin I as representative structures of betalains are shown in Figure 8. Betalains are red at pH 6–7, but instability with light and heat limits the application of betalains. Therefore, they can be used as a colorant for food products with a short shelf life and food stored at low temperature in opaque packings, such as ice cream, frozen desserts, and yogurts [96]. Betanin (E-number E162, CI Natural Red 33) as the main betalain component has been widely used as a colorant for food products, such as ice creams, yogurts, cake mixes, soft drinks, and gummy candies [97,98]. In the application of betanin as a food colorant, a content of less than 50 mg/kg can provide the most ideal color [97].

Indigo as an ancient natural dye is extracted from tropical plants, woad (*Isatis tinctoria*), and true indigo (*Indigofera tinctoria*) [99,100], and it is a remarkably stable blue dye and has a long history of use in textiles. The bis(indole) indigotin is responsible for the blue color of indigo-based dyes [101] and a new class of indigoids has been discovered [102]. FD&C blue no. 2 is its disulfonate sodium salt and has been used in food and cosmetic industries [103]. Synthetic indigo dyes contain only one pigment, indigo, and natural indigo dyes also contain indirubin, which have anti-inflammatory and anti-tumor effects [104]. Additionally, betalains and indigo dyes are widely used in medicine and other fields because of their extensive biological activities. In addition to these typical alkaloid dyes, some potential new alkaloid dyes have been isolated from microorganisms and plants in recent years (Table 2). Even though studies have shown that they show some pharmacological activities, their safety still needs further study.

#### 2.3.5. Quinones

**Figure 9:**
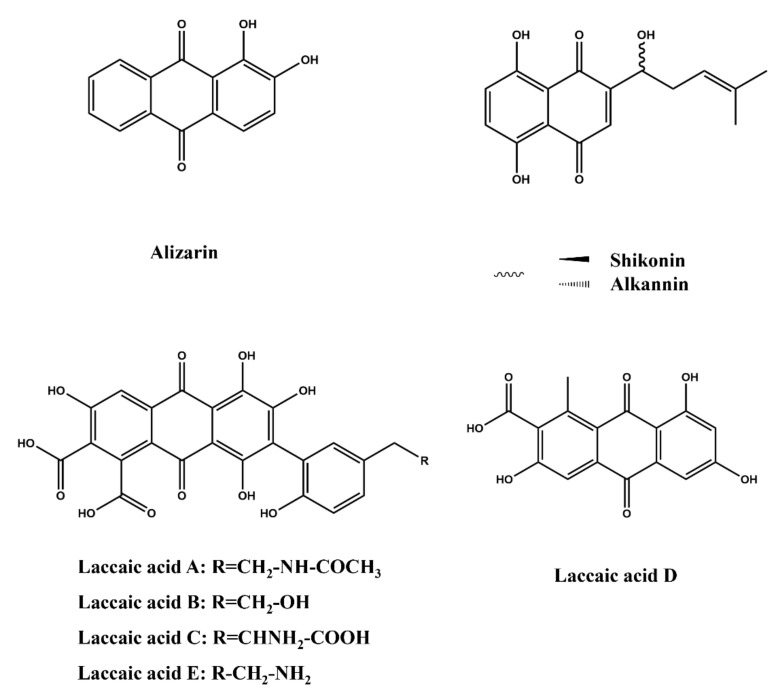
Structure of quinone dyes including alizarin, skikonin/alkannin, laccaic acid A/B/C/E, and laccaic acid D.

Alizarin, 1,2-dihydroxy-9,10-anthraquinone (Figure 9), is the principal component of the natural dye extracted from madder root (Rubia tinctorum) [105]. It is a type of anthraquinone fluorescent dye [106]. Because of their structural features, the anthraquinone derivatives can easily and closely interact with DNA molecules [107]. Therefore, they confer potential anti-tumor capabilities to these tricyclic planiform compounds because of the function of inducing cell apoptosis [108]. Alizarin has been applied in colorimetric indicators of PH, glucose, and others, for example, alizarin complexone functionalized mesoporous silica nanoparticles as smart nanoformulations which could be used in optical diagnosis, individualized treatment, and noninvasive monitoring of diabetes management [109].

Alkannin and its enantiomer shikonin are valuable natural dyes found in purple-colored roots of red gromwell (*Lithospermum erythrorhizon*) [110]. Recently, shikonins/alkannins have been discovered to exhibit a range of pharmacological properties and could be used as drug scaffolds to design a set of derivatives [111], which has excellent prospects in drug development. Furthermore, the components of lac dyes laccaic acid A, laccaic acid B, laccaic acid C, laccaic acid D, and laccaic acid E are also quinone dyes (Figure 9). In the last decade, marine resources and microorganism resources were the main sources of new quinone dyes, which suggested new directions and strategies for developing natural dyes. However, the toxicity and pharmacological activities of these new natural dyes need to be further studied so that they can be applied in the market.

### 2.4. Pharmacological Activities of Natural Dyes and Related Mechanisms

**Figure 10:**
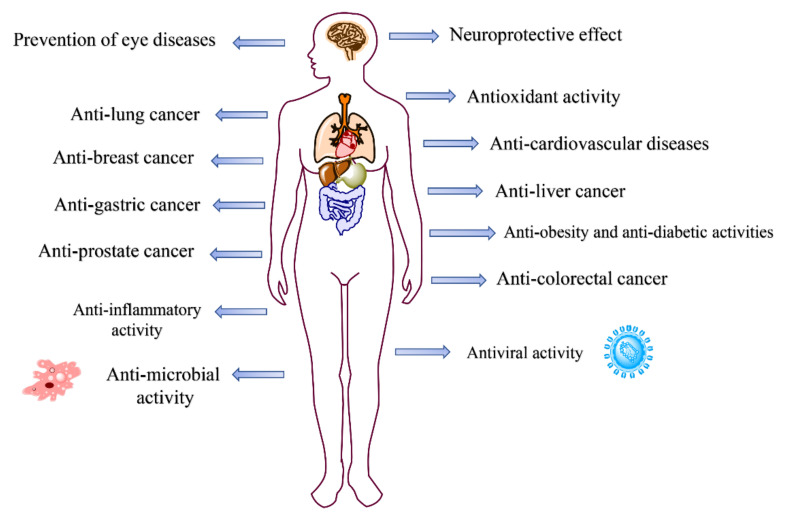
The pharmacological activities of natural dyes.

The pharmacological activities and related mechanisms of natural dyes have been studied extensively. The health benefits of carotenoids, anthocyanins, curcumins, and betalains were mainly discussed in recent years, including antioxidant activity, anti-inflammatory activity, anti-cancer activity, anti-cardiovascular disease activity, anti-obesity and anti-diabetic activity, anti-microbial activity, anti-viral activity, and neuroprotective effect (Figure 10).

#### 2.4.1. Antioxidant activities

Oxidative stress is caused by excessive production of reactive oxygen species (ROS), which leads to damage to cellular proteins, lipids, and DNA, and cell necrosis and apoptosis occur when the antioxidant defense system cannot eliminate ROS [112]. Oxidative stress might lead to many diseases including cardiovascular diseases, neurodegenerative diseases, obesity/diabetes, and cancer, and even be associated with human lifespan [113,114,115]. Nowadays, natural dyes such as carotenoid and polyphenol dyes are considered excellent antioxidants in nutraceutical and pharmaceutical fields. Carotenoids with provitamin activity can effectively scavenge ROS and reduce oxidative stress in the human body. In particular, astaxanthin showed excellent antioxidant activity in free radical scavenging, singlet oxygen quenching, inducing the antioxidant enzymeparaoxonase-1, enhancing glutathione concentrations, and preventing lipid peroxidation [116]. In the human umbilical vein endothelial cell (HUVEC) model, astaxanthin generated small amounts of ROS to activate the Nrf-2/HO-1 antioxidant pathway. However, it has been demonstrated that β-carotene at high doses shows pro-oxidant effects because it could produce radical ions that might damage cells [117]. In cellular and animal models, anthocyanins have been shown to reduce the generation of ROS and protect from oxidative damage, thereby preventing other diseases [118,119]. Anthocyanins as antioxidants could scavenge free radicals, enhance antioxidant enzyme activity, suppress oxidative stress via clearance of ROS, and sustain the level of GSH and the glutathione antioxidant defense system [120,121,122,123]. The antioxidant activity of curcumin was realized by inhibition of serum malondialdehyde, up-regulating transcription and expression levels of antioxidant enzymes and improving mitochondrial function [124,125]. Some mechanisms of antioxidant activity are shown in Table 3, showing that natural dyes can scavenge free radicals, reduce the generation of ROS, inhibit lipid peroxidation and malondialdehyde (MDA), and up-regulate transcription and expression levels of antioxidant enzymes including total superoxide dismutase (SOD), total catalase (CAT), and glutathione peroxidase (GPx). Antioxidant activity is considered to be an important mechanism for the treatment and prevention of other diseases, so it will be mentioned for other diseases.

#### 2.4.2. Anti-Inflammatory Activities

Growing evidence suggests that inflammatory responses play a critical role in the development and progression of major human diseases [134]. Oral administration of zeaxanthin could ameliorate acetic acid-induced colitis by antioxidative effects and modulation of pro-inflammatory cytokines. Zeaxanthin suppressed tumor necrosis factor-alpha (TNF-α), interferon-gamma (IFN-γ), interleukin-6 (IL-6), interleukin-1 beta (IL-1β), and nuclear transcription factor kappa B (NF-κB) levels, and inhibited nitric oxide synthase (iNOS) and cyclooxygenase-2 (COX-2) protein expression [130]. Oral administration with β-carotenoid ameliorated ulcerative colitis-associated local and systemic damage in mice by acting on multiple targets such as NF-κB, COX-2, STAT3 IL-17, nuclear erythroid 2 (NF-E2)-related factor 2 (Nrf2), matrix metalloproteinase-9 (MMP-9), and connective tissue growth factor [135]. Studies have found that the anti-inflammatory mechanisms of astaxanthin involve multiple signaling pathways including PI3K/AKT, Nrf2, NF-κB, ERK1/2, JNK, p38 MAPK, and JAK-2/STAT-3, and the anti-inflammatory effects showed preventive effects on a variety of diseases [136]. Malvidin 3,5-diglucosid as an anthocyanin could reduce inflammation symptoms through reducing NO production and reducing the induction of pro-inflammatory cytokines such as IL-1β, TNF-α, and IL-6 in lipopolysaccharide (LPS)-induced RAW264.7 macrophages [137]. The anti-inflammatory action effect of anthocyanins can be attributed primarily to their antioxidant properties. Anthocyanins extracted from Trifolium pratense (red clover) inhibited the expression of genes such as TNF-α, IL-1β, iNOS, COX-2, and monocyte chemoattractant protein (MCP-1) and translocation of the p65 subunit of NF-κB into the nucleus [138]. In addition to down-regulation of the redox-sensitive nuclear NF-κB signaling pathway, the mitogen-activated protein kinase pathways also appeared to play a role [139]. Curcumin exerts anti-inflammatory effects by regulating inflammatory signaling pathways and inhibiting the production of inflammatory mediators. Curcumin exerted its anti-inflammatory effect by inhibiting TLR4 expression, the phosphorylation of ERK, JNK, p38, and NF-κB in macrophages and TLR4-MAPK/NF-κB pathways were involved [140]. The NF-κB signal pathway is the principal inflammatory signaling pathway, and it reduces the expression of inflammatory cytokines including IL-1β, IL-6, TNF-α, COX-2. Carotenoids, anthocyanins, and curcumin all regulate this pathway to achieve anti-inflammatory effects, and natural dyes can be used as food supplements to treat inflammation and a variety of inflammation-related diseases.

#### 2.4.3. Anti-Cancer Activities

The incidence of cancers continuously increased in the last few years and cancers have overtaken cardiovascular diseases as the leading cause of death in some high-income countries [141]. Cancer of the endocrine, digestive, urinary, and immune systems includes breast cancer, liver cancer, gastric cancer, colorectal cancer, prostate cancer, lung cancer, skin cancer, etc. [142]. Natural dyes have shown anti-cancer activities against several types of cancer (Table 4). Carotenoids including β-carotene, lycopene, crocin, astaxanthin, and fucoxanthin have shown anti-proliferative and pro-apoptotic actions against various cancers. For example, astaxanthin has been reported to inhibit the proliferation of breast cancer cells by modulating different signaling pathways and molecular targets such as inhibition of cellular migration and cell number, suppressing expression levels of pontin, mutp53, Oct4, and Nanog, and activation of Bax/Bcl2, cleaved caspase-3, and cleaved caspase-9 as well as the phosphorylation of ERK1/2, JNK, and p38. Anthocyanins play a potential role in preventing and treating cancer [143,144,145]. Anthocyanin extracts and anthocyanins, such as cyanidin-3-glucoside (C3G), peonidin-3-glucoside (Pn-3-G), malvidin-3-glucoside (M3G), delphinidin-3,5-*O*-diglucoside (D-3-5-D), cyaniding-3-rutinoside (C3R), pelargonidin-3-glucoside (Pg-3-G), cyanidin-3-xylosylutinoside (C3XR), and proanthocyanidins, have been reported have inhibitory effects on breast cancer, colorectal cancer, liver cancer, lung cancer, and prostate cancer. For example, anthocyanin extracts from purple potato can suppress colon tumorigenesis by suppressing the Wnt/β-catenin signaling pathway and enhancing mitochondrion-mediated apoptosis [146]. M3G as an adjuvant ingredient or nutritional supplement can prevent liver cancer by inhibiting proliferation, migration, and invasion-related pathways and promoting the apoptosis of liver tumor cells [147]. In addition, grape seed proanthocyanidins showed a radioprotective effect on normal lung cells and were considered an ideal radioprotective drug for lung cancer patients treated with radiotherapy [148]. Curcumin is a promising candidate for prevention and treatment of several cancers. The anti-cancer activities of curcumin involve different signaling pathways and molecular targets including modulation of growth factors, enzymes, transcription factors, kinases, and inflammatory cytokines, and up-regulating pro-apoptotic and down-regulating anti-apoptotic proteins [149]. The anti-lung cancer activity of curcumin has been reported and curcumin could suppress cell proliferation and induce apoptosis via modulating the JAK2/STAT3 signaling pathway, PI3K/Akt signaling pathway, and Wnt/β-catenin pathway [150,151,152,153]. Therefore, natural pigments can inhibit the proliferation of cancer cells and induce apoptosis in colon cancer, breast cancer, lung cancer, liver cancer, gastric cancer, prostate cancer, etc., and can be considered nutritional supplements and food additives.

#### 2.4.4. Anti-Obesity and Anti-Diabetic Activities

The WHO estimates that by 2025, approximately 167 million people will become less healthy because they are overweight or obese (https://www.who.int/news/item/04-03-2022-world-obesity-day-2022-accelerating-action-to-stop-obesity (accessed on 1 April 2022)). Obesity occurs when excess adipose tissue accumulates in the body, which can result in metabolic syndrome, including type 2 diabetes, hypertension, and dyslipidemia [207]. (3R,3′R)-Astaxanthin could be a supplement to prevent weight gain, reduce plasma and liver triacylglycerol, and decrease plasma and liver total cholesterol [208]. At the same time, carotenoids could regulate gut microflora to reduce the incidence of obesity [208,209]. Lutein showed a preventive effect against cardiac and renal injury in STZ-induced hyperglycemic rats by altering antioxidant enzyme activities [129]. In anti-diabetic activity, astaxanthin could attenuate STZ-induced diabetes by decreasing blood glucose and total cholesterol levels, and increasing blood levels of high-density lipoprotein cholesterol (HDL-C) in a dose-dependent manner [210]. Anthocyanins have shown anti-obesity effects through multiple mechanisms including inhibiting lipid absorption, regulating lipid metabolism, increasing energy expenditure, suppressing food intake, and regulating gut microflora [211]. Anthocyanins could suppress lipid accumulation in adipocytes [212] and reduce high-fat diet-induced metabolic damage [213]. Body mass index and body weight were reduced when anthocyanin supplementation was 300 mg/d or less for 4 weeks [214]. Cyanidin 3-caffeoyl-p-hydroxybenzoylsophoroside-5-glucoside as an anthocyanin isolated from purple-fleshed sweet potato showed hypoglycemic effects by specifically suppressing hepatic glucose output [215]. C3G from black soybeans showed anti-diabetes effects by inducing the differentiation of 3T3-L1 preadipocytes into smaller and insulin-sensitive adipocytes [216]. Curcumin has been used as a pharmacological traditional medicinal agent in Ayurvedic medicine for about 6000 years. The anti-obesity mechanisms of curcumin are associated with the enzymes, energy expenditure, adipocyte differentiation, lipid metabolism, gut microflora, and anti-inflammatory potential [217]. Betacyanins purified from *Hylocereus undatus* peel could ameliorate obesity and insulin resistance in high-fat diet-fed mice [218]. Natural pigments could be considered as dietary supplements to prevent and ameliorate obesity and type 2 diabetes.

#### 2.4.5. Anti-Cardiovascular Disease Effects

Cardiovascular diseases (CVDs) are a group of disorders that affect the heart and blood vessels and represent the leading cause of morbidity and mortality worldwide [219]. Oxidative stress and inflammation play an important role in CVDs. It has been suggested that carotenoids with antioxidant activity could prevent and ameliorate CVDs by suppressing oxidative stress and mitigating inflammatory responses [220]. Lutein, a major carotenoid, showed a protective effect in a cardiac failure rat model by improving cardiac morphology, antioxidant status via positively regulating the Nrf2/HO-1 signaling pathway, and reducing inflammatory markers (IL-1β, IL-6, TNF-α, NF-κB, p65) and apoptotic markers (caspase-3 and caspase-9) [221]. Data from several epidemiological studies have reported an inverse correlation between anthocyanin intake and risk of CVDs or CVD-related mortality. Higher habitual anthocyanin intake was also inversely associated with a risk of total myocardial infarction in premenopausal women [222] and nonfatal myocardial infarction in men [223]. Molecular mechanisms of action of anthocyanins are complex and include modulation of gene expression, cell signaling, and miRNA expression [224]. Curcumin has been found to ameliorate various CVDs such as atherosclerosis, cardiac hypertrophy, cardiac fibrosis, heart failure, myocardial infarction, and ischemia by multiple mechanisms and modulating multiple signaling pathways [225]. For example, recent research has found that curcumin shows a significant protective effect in myocardial ischemia–reperfusion by activating the PI3K/AKT/mTOR signaling pathway and inhibiting inflammation, apoptosis, and oxidative stress [226]. Betalain treatment protected hearts from failing via microRNA-mediated activation of the anti-inflammatory signaling and restoring the matrix protein modulation [132].

#### 2.4.6. Anti-Microbial Activity

Red cabbage and sour cherry pomace anthocyanin extracts show anti-microbial effects on *Escherichia coli*, *Staphylococcus aureus*, *Listeria monocytogenes*, *Salmonella Typhimurium*, and *Bacillus cereus* [227]. Curcumin has a broad spectrum of anti-bacterial actions against a wide range of bacteria [228]. Curcumin and its derivatives (curcumin monoglucoside, curcumin diglucoside) possess strong anti-microbial properties against *Streptococcus pneumoniae*, even in penicillin-resistant strains. Curcumin showed anti-bacterial activity against tested strains of methicillin-resistant *S. aureus* because of increased membrane permeability and DNA fragmentation [229]. The efficacy of curcumin against *Helicobacter pylori* has been studied and the potential mechanism is down-regulation of IL-17 through the induction of indoleamine 2,3-dioxygenase in H. pylori-infected human gastric mucosa [230]. The anti-fungal activity of curcumin has also been reported and curcumin could inhibit biofilm formation and filamentation of *Candida albicans*. In anti-microbial photodynamic therapy, chlorophyll a and chlorophyll b exhibited high anti-microbial activity under irradiation [231]. Additionally, betalains have shown inhibitory effects against Gram-negative bacteria such as *Pseudomonas aeruginosa* and *S. Typhimurium*, among others, and Gram-positive bacteria, such as *S. aureus*, *Enterococcus faecalis*, and *L. monocytogenes* [232,233]. Dyes extracted from *Rhodotorula glutinis* could effectively inhibit the growth of *B. cereus*, *Salmonella enteritidis*, and *E. coli* [234]. Chaetoviridide A and Chaetoviridide B are new dye compounds isolated from the deep-sea fungus *Chaetomium* sp. NA-S01-R1, and they showed anti-bacterial activities against *Vibrio rotiferianus* and *Vibrio vulnificus* [235]. In addition, the applications of natural dyes isolated from fungi in healthcare have been explored, and silk sutures treated with an optimum concentration of natural fungal dye could inhibit the growth of *S. aureus* and *E. coli* [236]. Therefore, natural dyes play a potential role in anti-microbial activity against different pathogenic bacteria and have a bright prospect in healthcare applications.

#### 2.4.7. Anti-Viral Activities

The carotenoids isolated from haloalkaliphilic archaeon *Natrialba* sp. M6 exhibited significantly stronger activity in eliminating hepatitis C virus (HCV) and hepatitis B virus (HBV) in infected human blood mononuclear cells than currently used drugs. This anti-viral activity may be attributed to its inhibitory potential against HCV RNA and HBV DNA polymerases, which thereby suppresses HCV and HBV replication, as indicated by a high viral clearance % in the treated cells [237]. A marine carotenoid, siphonaxanthin from Codium fragile, showed significant anti-viral activity with an IC_50_ of 87.4 µM against SARS-CoV-2 pseudovirus entry, and was predicted to have relatively low acute toxicities [238]. Anthocyanin fractions of strawberry, raspberry, bilberry, and lingonberry showed strongly anti-viral effects against influenza virus A/H3N2 through inhibiting the replication of the virus [239]. Delphinidin, belonging to the anthocyanin family, has shown inhibitory effect against HCV by a new mechanism, alteration of the viral particle structure, that impairs its attachment to the cell surface [240]. The anti-viral activity of curcumin has been widely studied, and the main mechanisms include direct interference with viral replication machinery and suppression of cellular signaling pathways essential for cellular replication, such as PI3K/Akt, NF-κB [241]. In Vero cells infected with EV71, the addition of curcumin significantly suppressed the synthesis of viral RNA, the expression of viral protein, and the overall production of viral progeny [242]. A recent study showed that curcumin inhibited in vitro SARS-CoV-2 infection in Vero E6 cells by affecting the SARS-CoV-2 replicative cycle and curcumin exhibited a virucidal effect with a variant/strain-independent anti-viral effect and immune-modulatory properties [243]. Natural dyes with anti-viral activity might play a potential role in the development and progression of COVID-19, which should be explored further.

#### 2.4.8. Neuroprotective Effect

Altered amyloid precursor protein (APP) processing potentiates the aggregation of glycation products, and amyloid-β (Aβ) toxicity is a key pathogenic feature of Alzheimer’s disease (AD) [244]. Carotenoids including cryptocapsin, cryptocapsin-5,6-epoxide, and zeaxanthin showed anti-amyloidogenic potential by preventing the formation of the fibril and through disruption of the Aβ aggregates [245]. Lutein protected dopaminergic neurons by enhancing antioxidant defense and diminishing mitochondrial dysfunction and apoptotic death, suggesting the potential benefits of lutein for Parkinson’s disease treatment [246]. Natural dietary supplementation of anthocyanins could ameliorate neurodegeneration and memory impairment in a mouse model of Alzheimer’s disease. Anthocyanins as a potent antioxidant neuroprotective agent reduced AβO-induced neurotoxicity in HT22 cells via the PI3K/Akt/Nrf2 signaling pathway and improved memory-related pre- and postsynaptic protein markers and memory functions in APP/PS1 mice [247]. Bilberry anthocyanin consumption was considered to reverse AD-induced cognitive disfunction, decrease hippocampal neuroinflammatory responses, and induce phagocytosis of microglia to beta-amyloid protein plaques by regulating the CD33/TREM2/TYROBP signaling pathway in microglia [248]. Pelargonidin belongs to the anthocyanins and has been found to improve Aβ (25–35)-induced memory deficit through mitigation of oxidative stress, cholinergic dysfunction, and astrocyte reaction [249]. The effect of curcumin on AD involves multiple signaling pathways such as anti-amyloid and metal iron chelating properties, antioxidation and anti-inflammatory activities [250], and curcumin treatment protected rat PC12 cells from Aβ (25–35)-induced reduction in cell viability, apoptosis, the release of LDH, and MDA production [251]. In AlCl*_3_*-induced AD rats, betalain ameliorated AD by modulating oxidative stress and the NF-κB signaling pathway [252]. Therefore, natural dyes could be a potent dietary supplement with antioxidant and neuroprotective effects.

#### 2.4.9. Biological Effects of Dyes Regarding Illumination Conditions

Different from other compounds, dyes have the unique feature that the biological effects are affected by illumination conditions (strict dark, ambient light, or controlled illumination). Photodynamic therapy (PDT) is a promising new treatment which uses suitable photosensitizers, appropriate wavelengths of light, and oxygen to kill cancer cells and microorganisms [90]. In recent years, the application of natural dyes as photosensitizers in PDT has been studied. Erythrosine combined with C3G as photosensitizers in PDT could eliminate *Porphyromonas gingivalis* biofilms [253]. PDT using purpurin (an anthraquinone pigment) could effectively inhibit the growth of triple negative breast cancer cells both in vitro and in vivo [254]. Blue light-activated curcumin markedly damaged membrane permeability, resulting in cell death of *S. aureus* [255]. A clinical trial suggested oral curcumin together with visible light might be a new therapeutic method for moderate to severe plaque psoriasis [256]. Chlorophylls and derivatives as photosensitizers in PDT could be used to treat acne vulgaris and microbial infection [231,257,258,259]. In therapeutic PDT, the photosensitizers should absorb between 600 and 800 nm, and natural dyes might show potential as photosensitizers, which is worthy of further study.

In fact, there are some debates about the biological activity of curcumin because it is an unstable, reactive, nonbioavailable compound, which is also considered a PAINS medication [260]. Some studies have found no significant difference between curcumin and placebo [261], but some pharmacological activities of curcumin have been validated in animal experiments and clinical trials in recent years [262,263,264,265]. Many factors affect the activity of curcumin. The biggest problem is its poor water solubility, low absorption, and fast metabolization and clearance. Therefore, curcumin is not necessarily ineffective, which might be due to the low bioavailability. Recently, the interaction between gut microbiota and curcumin was hypothesized to explain how curcumin directly exerts its regulatory effects on the gut microbiota, thus explaining the paradox between its low systemic bioavailability and its wide pharmacological activities [266]. Interestingly, light irradiation could enhance the biological effects of curcumin. Low-dose curcumin plus visible light exposure could significantly inhibit metastatic processes of renal cell carcinoma [267], and light exposure might also enhance its efficacy in bladder cancer cell lines [268]. Exposure to light also enhanced its anti-microbial capacity because of curcumin phototoxicity in bacterial cells [269]. Therefore, although light affects the stability of some dyes, whether light exposure can enhance the pharmacological activities of other dyes is still a problem. The biological effects of the dyes regarding the illumination conditions should be studied. Even so, it is necessary to improve the stability and bioavailability of natural dyes, and nanoscale formulations are effective strategies and discussed in the next section.

### 2.5. Challenges and Potential of Natural Dyes

Although a variety of natural dyes have shown preventive and therapeutic effects on a variety of diseases, there are still some challenges in practical application. (1) Stability: Usage of this colorant in other products is limited by its poor stability to heat, light, and pH conditions. (2) Water solubility and bioaccessibility: Some natural dyes including carotenoids and curcumin have poor water solubility that limits their oral administration and decreases their bioavailability. (3) Resource constraints and extensive use: Some natural plant dyes will be limited by seasons and resources. In order to solve those problems, some strategies have been put forward.

#### 2.5.1. Resources

According to the published literature, the study of marine natural products has increasingly captured the attention of scientists in recent years. Some new natural dyes including carotenoids and quinone dyes have been isolated from marine resources [23,24,51,77]. Additionally, new sources of natural dyes have been found in marine environments, such as a fast-growing strain of *Chlorella saccharophila* and a new variety of the ridgetail white prawn [270,271]. Marine resources show great potential for screening for new natural dyes and high-yield dye sources. In contrast to other resources, microorganisms have enormous advantages including rapid growth, easy processing, and independence from weather conditions. Apart from colorants, some bacterial, fungal, and microalgal dyes possess many biological properties such as antioxidant, anti-microbial, and anti-cancer activities [90,272]. Microorganisms are an abundant source of novel bioactive compounds and, unlike higher organisms, they are a source of easily renewable resources that give rise to production with a potentially greater yield [27,273]. Some pigment-producing microorganisms are easy to culture and have low production cost, so they are becoming more and more important application objects in the production of natural dyes, and have very broad application prospects. The optimized culture conditions could accelerate biosynthesis of the dyes and enhance dye production [272,274]. Therefore, exploration of marine resources and microorganism dyes is necessary.

#### 2.5.2. Biotechnology

As described above, microorganisms have great potential in the development of natural dyes. In recent years, metabolic and genetic engineering approaches have been made to modify or introduce particular pathway genes into microorganisms to increase production of natural dyes, especially carotenoids and anthocyanins [275,276]. In recent years, it has been reported that heterologous genes were transferred into *E.* *coli* to synthesize carotenoids. *E. coli* has been engineered to produce various carotenoids, including lycopene, carotene, astaxanthin, and crocin [277]. Park et al. introduced heterologous crt genes (crtE, crtY, crtI, crtB, and crtZ) from *Pantoea ananatis* and the truncated BKT gene (trCrBKT) from *Chlamydomonas reinhardtii* to construct the astaxanthin biosynthetic pathway, and enhanced production of astaxanthin [278]. *E. coli* has been considered the most suitable for anthocyanin production in previous studies, and some anthocyanins were obtained from this microbial cell factory, such as cyanidin 3-*O*-glucoside and 3′-*O*-methylated and peonidin 3-*O*-glucoside [279,280]. *Saccharomyces cerevisiae* has also been used to produce anthocyanins in recent years. Eichenberger et al. engineered *S. cerevisiae* for de novo production of the three basic anthocyanins, pelargonidin-3-*O*-glucoside, cyanidin-3-*O*-glucoside, and delphinidin-3-*O*-glucoside [281]. An *S. cerevisiae–S. cerevisiae* co-culture platform was designed to manufacture two anthocyanidins in flask-scale culture [282]. Biotechnology makes the production of anthocyanin no longer dependent on plants alone. With optimizing the culture and advances in biotechnology, microbial cell factories are the most promising system to increase the yield of natural dyes for commercial and industrial purposes.

#### 2.5.3. Efficient Extraction and Separation Strategy

In the actual development and application of natural dye, high-yield, lower-cost, and environment-friendly technology is also very important. In recent years, some novel extraction techniques have shown these advantages.

Ultrasound-assisted extraction is an effective method to extract natural dyes, and it has been widely used in the extraction of anthocyanins and carotenoids because of shorter extraction time, higher efficiency, and low solvent volumes [283,284]. The microwave-assisted extraction process is economical due to shorter extraction time and less solvent consumption. This method has been used to extract natural dyes from plants [285,286]. Supercritical fluid extraction is not only an advanced extraction technology for natural products, but also an environmentally friendly technology. Recent studies used supercritical CO_2_ to extract carotenoids and anthocyanins [287]. Regardless of the extraction method used, the optimized conditions should be screened and used to improve yield. In the separation of natural dyes, high-speed countercurrent chromatography (HSCCC) as a chromatographic technique of the liquid-liquid type with large sample recovery and low loss showed a bright prospect to separate natural dyes [288,289]. In fact, those techniques can be used in combination. In order to extract anthocyanins more efficiently, a novel procedure of ultrasound-assisted deep eutectic solvent extraction was proposed, and the HSCCC method was involved in the separation and purification of anthocyanins [289].

#### 2.5.4. Improvement of Dye Stability

##### Co-Pigmentation

Co-pigments form noncovalent complexes with anthocyanins and have been used to enhance color and stability. Different co-pigments could change the properties of pigments by hyperchromic and bathochromic shifts [290]. Co-pigmentation of anthocyanins and phenolic compounds and plant extracts has been reported. Rosmarinic acid, syringic acid, and catechin showed significant hyperchromic effects for black chokeberry (*Aronia melanocarpa*) anthocyanin [291]. Molecular modeling results showed that multiple co-pigments may intensify the color of anthocyanins more than individual ligands, and phenolic acid–flavonol–anthocyanin could be used as promising food red-colorants [292]. Blueberry wines showed higher alcohol and titratable acidity, and lower sugar content by addition of caffeic acid, syringic acid, and quercetin co-pigments during fermentation [293]. Additionally, herbal extracts and plant extracts improved the stability of anthocyanins, hyperchromic effect, and color density [291,294]. Piperine could be used to increase the bioavailability of curcumin because of the molecular interactions of curcumin and piperine [295]. However, co-pigmentation depends on the intermolecular interaction between co-pigments and anthocyanins, and different co-pigments should be selected for different dyes.

##### Encapsulation

Encapsulation systems have been used to increase the solubility, chemical stability, pharmacological activity, and bioavailability of natural dyes. Maltodextrin, lipid-based nanocarriers including nanoliposomes, nanoemulsions and solid lipid nanoparticles [296], biopolymer-based nanocarriers such as proteins [297], carbohydrates, and chitosan [298], gold nanoparticles [299], and clay minerals [293] have been studied.

Liposomes have shown many advantages and attracted extensive attention because of their good stability, nontoxicity, water-solubility, good cell compatibility, and targeted delivery [300]. Based on the above advantages and colorless liposomes and the instability of natural dyes, liposomes are widely used to encapsulate natural dyes to improve their stability and targeting delivery. Carotenoids including lutein, β-carotene, lycopene, and canthaxanthin were encapsulated in liposomes and the delivery systems improved the antioxidant activity of carotenoids in a DPPH-scavenging assay and ferric reduction capacity assay [301]. A study demonstrated that propolis–lycopene nanoemulsions could protect skin against UVA radiation and confer better therapeutic effects [302]. In another study, lycopene was loaded on nanostructured lipid carriers and solid lipid nanoparticles [303], and they had a significantly improved effect on neuronal protection [296]. To protect the anthocyanin from adverse external conditions, liposomes were prepared with a supercritical carbon dioxide process and had improved efficacy and potential application in food and nutraceuticals [304]. In addition, anthocyanin-loaded liposomes could effectively enhance the stability of anthocyanin, antioxidant activity, and skin permeability [305]. Shikonin-loaded liposomes were prepared with 1,2-dipalmitoyl-phosphatidylcholine and egg phosphatidylcholine lipids, which could reduce the side effects, enhance selectivity to cancer cells and solubility, and protect shikonin from internal biotransformations and oxidization [306]. Curcumin-loaded liposomes have been extensively studied to solve the disadvantages including poor aqueous solubility and low bioavailability [307]. Betanin-nanoliposomes could improve the stability and antioxidant activity of betanin. Gummy candies using betanin-nanoliposomes showed no differences in the sensory properties [308].

Therefore, liposomes are considered to be ideal models for encapsulating natural dyes, which can improve stability, enhance the bioavailability, and achieve targeted delivery.

## 3. Conclusions and Future Prospects

Natural dyes widely exist in plants, anmimals and microorganisms, and show multiple healthy effects including antioxidant, anti-inflammatory, anti-cancer, anti-microbial, and anti-viral effects, and prevention and treatment of a variety of diseases, including diabetes, obesity, cardiovascular and cerebrovascular diseases, eye diseases, and nervous system diseases. Many natural dyes have been developed as drugs and functional foods. Some natural dyes have been approved by the FDA as food additives, such as curcumin and phycocyanin. Natural dyes show great advantages in safety, but there are still problems such as stability, solubility, and economic applicability. Furthermore, the unique feature that the biological effects of dyes are affected the illumination conditions (strict dark, or ambient light, or controlled illumination) should be paid attention. Some new strategies for these problems have been proposed. Natural dyes have great potential for the discovery of new drugs and functional food products. In terms of cost, marine resources and microorganisms are considered potential resources of natural dyes in the future. Some new technologies could be used for the production, extraction, and separation of natural dyes to improve the yield of natural dyes, such as metabolic and genetic engineering approaches, ultrasound-assisted extraction, and HSCCC. Co-pigments and encapsulation systems have been studied extensively and proven to improve the hyperchromic effect, stability, solubility, and bioavailability. Red, yellow, and blue dyes can be screened or modified from natural dyes, and other colors can be deployed in different proportions.

In conclusion, natural dyes play an important role in food, medicine, textile, and other industries, which make human life more colorful. This review classifies natural dyes by structural features and summarizes the research progress on natural dyes in the last ten years, including some of the newest dyes, pharmacological activities, and promising strategies for developing natural dyes. The review provides new insight for further development and potential applications of natural dyes.

## Figures and Tables

**Table 1:** Terms used in the search strategy.

Electronic Database	Search and Terms
Web of Science PubMed	#1 (“Natural dye” OR “Natural pigment” OR “Natural colorants”) AND (“Carotenoids” OR “Anthocyanins” OR “Curcumin” OR “Chlorophylls” OR “Alkaloid” OR “Quinone”) #2 “New” And “Pigment” AND (“Carotenoids” OR “Anthocyanins” OR “Curcumin” OR “Alkaloid” OR “Quinone”) AND “Isolated” #3 (“Carotenoids” OR “Anthocyanins” OR “Curcumin” OR “Betalain”) AND (“Antioxidant” OR “Inflammatory” OR “Anti-cancer” OR “Cancer” OR “Anti-bacterial” OR “Antimicrobial” OR “Obesity” OR “Anti-obesity” OR “Diabetes” OR “Cardiovascular” OR “Anti-viral” OR “Neuroprotective” OR “Alzheimer’s disease”) #4 (“Extraction” OR “Isolation” OR “Extracted” OR “Isolated”) AND (“Carotenoids” OR “Anthocyanins”) AND “New method”

**Table 2:** Several newly isolated and identified natural dyes in the last 10 years.

Category	Compounds	Source	Ref.
Carotenoids	6′-Epimonadoxanthin	Rosary goby (*Gymnogobius castaneus*)	[51]
	3′-Deoxycapsorubin	Red mamey (*Pouteria sapota*)	[52]
	3,3′-Dideoxycapsorubin	Red mamey (*Pouteria sapota*)	[52]
	Methyl 5-glucosyl-5,6-dihydro-apo-4,4′-lycopenoate	*Planococcus maritimus* strain iso-3	[53]
	Diapolycopenedioc Acid Xylosylesters A/B/C	*Rubritalea squalenifaciens*	[53]
	13Z-zeaxanthin dipalmitate	Wolfberry	[54]
Anthocyanins	Malvidin-3-(p-coumaroyl)-rutinoside-5-glucoside	Transgenic Del/Ros1 tomato fruit	[55]
	Malvidin-3-(feruloyl)-rutinoside-5-glucoside	Transgenic Del/Ros1 tomato fruit	[55]
	Petunidin-3-(cis-p-coumaroyl)-rutinoside-5-glucoside	Tomato cultivar Indigo Rose	[56]
	Malvidin-3-(cis-p-coumaroyl)-rutinoside-5-glucoside	Tomato cultivar Indigo Rose	[56]
	Petunidin-3-(trans-p-coumaroyl-rhamonside)-glucoside-5-glucoside	Tomato cultivar Indigo Rose	[56]
	Malvidin-3-(p-methoxy-trans-coumaroyl)-rutinoside-5-glucosid	Tomato cultivar Indigo Rose	[56]
	Delphinidin 3-*O*-a-l-rhamnopyranosyl-(1→6)-b-d-glucopyranoside-30-*O*-b-d-glucopyranoside	Tamarillo fruit	[57]
	Cyanidin 3-[2′’-(6′’’-coumaroyl)-glucosyl]-glucoside	Nitraria tangutorum	[58]
	Pelargonidin-3-*O*-coumaroylglucoside	Mulberry (Morus moraceae) juice	[59]
	Delphinidin-3-*O*-coumaroylglucoside	Mulberry (Morus moraceae) juice	[59]
	Cyanidin 3-*O*-[2-*O*-(2-*O*-(4-*O*-(6-*O*-(4-*O*-(β-glucopyranosyl)-trans-caffeoyl)-β-glucopyranosyl)-trans-caffeoyl)-β-glucopyranosyl)-6-*O*-(trans-sinapoyl)-β-glucopyranoside]-5-*O*-[6-*O*-(malonyl)- glucopyranoside]	Purple-violet flowers of *Moricandia arvensis*	[60]
	5,7-Dimethylmalvidin 3-*O*-β-galactopyranoside	Blue Plumbago flower	[61]
	5,7-Di-methylpetunidin 3-*O*-β-galactopyranoside	Blue Plumbago flower	[61]
	5,7-Di-methyldelphinidin 3-*O*-β-galactopyranoside	Blue Plumbago flower	[56]
	5,7-Dimethylmalvidin 3-*O*-α-rhamnopyranoside	Blue Plumbago flower	[61]
	5,7-Dimethyldelphinidin 3-*O*-α- rhamnopyranoside	Blue Plumbago flower	[61]
	5,7-Dimethylpetunidin 3-*O*-α-rhamnopyranoside	Blue Plumbago flower	[61]
	petunidin 3-*O*-[6-*O*-(4-*O*-(4-*O*-cis-(β-d-glucopyranoside)-p-coumaroyl)-α-l-rhamnopyranosyl)-β-d-glucopyranoside] -5-*O*-[β-d-glucopyranoside]	Wild *Lycium ruthenicum* Murr.	[62]
	3-*O*-(6-*O*-α-l-Rhamnopyranosyl-β-d-glucopyranosyl)-7-*O*-(6-*O*-(4-*O*-(6-*O*-(E)-caffeoyl-β-d-glucopyranosyl)-(E)-caffeoyl)-β-d-glucopyranosyl) del-phinidin	Bluish-purple petals of Chinese bellflower (*Platycodon grandifloru*)	[63]
	3-*O*-(6-*O*-α-l-Rhamnopyranosyl-β-d-glucopyranosyl)-7-*O*-(6-*O*-(4-*O*-(6-*O*-(4-*O*-β-d-glucopyranosyl-(E)-p-coumaroyl)-β-d-glucopyranosyl)-(E)-caffeoyl)-β-d-glucopyranosyl) delphinidin	Bluish-purple petals of Chinese bellflower (*Platycodon grandifloru*)	[63]
	3-*O*-(6-*O*-α-l-Rhamnopyranosyl-β-d-glucopyranosyl)-7-*O*-(6-*O*-(4-*O*-(6-*O*-(4-*O*-β-d-glucopyranosyl-(E)-caffeoyl)-β-d-glucopyranosyl)-(E)-p-coumaroyl)-β-d-glucopyranosyl) delphinidin	Bluish-purple petals of Chinese bellflower (*Platycodon grandifloru*)	[63]
	Alatanin D	Purple yam (*Dioscorea alata* L.)	[64]
	Alatanin E	Purple yam (*Dioscorea alata* L.)	[64]
	Alatanin F	Purple yam (*Dioscorea alata* L.)	[64]
	Alatanin G	Purple yam (*Dioscorea alata* L.)	[64]
	Panaxidin A (pelaragonidin-4-vinylcatechol)	*Panax quinquefolius* L.	[65]
	Panaxidin B (pelargonidin-4-vinylphenol)	*Panax quinquefolius* L.	[65]
Alkaloid	Alstoscholarisine F/G	*Alstonia scholaris*	[66]
	Oryzadiamine C	Oryza sativa mutant	[67]
	Oryzadiamine A	Oryza sativa with yellow grain	[68]
	Rosellin A	Mushroom *Mycena rosella*	[69]
	Rosellin B	Mushroom *Mycena rosella*	[69]
	Ergopigment 8/9/10	*Claviceps purpurea*	[70]
	Katorazone	*Streptomyces* sp. IFM 11299	[71]
	2-(4-((3E,5E)-14-Aminotetradeca-3,5-dienyloxy) butyl)-1,2,3,4-tetrahydroisoquinolin-4-ol	*Fusarium moniliforme* KUMBF1201	[72]
	6′-*O*-malonyl-amaranthin	Callus culture of *Celosia cristata* L.	[73]
Quinone	Hypalocrinins A/B/C/D/E/F/G	Deep-sea crinoid *Hypalocrinus naresianus*	[24]
	5′-Hydroxytrypethelone	The mycobiont of lichen Trypethelium eluteriae Sprengel	[74]
	Gymnochrome A/H	Deep-sea crinoid *Hypalocrinus naresianus*	[23]
	1,4,6b,7,10-Pentahydroxy-1,2,6b,7,8,12b-hexahydroperylene-3,9-dione	Endophytic fungus *Alternaria tenuissima* SS77	[75]
	1,4,9,12a-Tetrahydroxy-12-methoxy-1,2,11,12,12a,12b-Hexahydroperylene-3,10-dione	Endophytic fungus *Alternaria tenuissima* SS77	[75]
	1,4,9-tri-hydroxy-1,2-Dihydroperylene-3,10-dione	Endophytic fungus *Alternaria tenuissima* SS77	[75]
	Alaternosides A/C	*Rhamnus alaternus* L	[76]
	6-Methoxy-rhodocomatulin 7-methyl ether	Australian sponge *Clathria hirsuta*	[77]
	3-Bromo-6-methoxy-12-desethyl- rhodocomatulin 7-methyl ether	Australian sponge *Clathria hirsuta*	[77]
	3-Bromo-6-methoxy-rhodocomatulin 7-methyl ether	Australian sponge *Clathria hirsuta*	[77]
	3-Bromorhodocomatulin 7-methyl ether	Australian sponge *Clathria hirsuta*	[77]
	Grandiquinone A	Leaves of *Tectona grandis*	[78]
	Phomopsanthraquinone	Fungus *Phomopsis* sp. PSU-MA214	[79]

**Table 3:** Mechanisms of some natural dyes for antioxidant activity.

Category	Compounds Name	Mechanism	Refs.
Carotenoids	Astaxanthin	Scavenged free radicals, quenched singlet oxygen, ↑ antioxidant enzyme paroxoanase-1, ↑ glutathione concentrations, ↓ lipid peroxidation.	[116]
		Activated the Nrf-2/HO-1 antioxidant pathway by generating small amounts of ROS in HUVEC model.	[126]
		↓ Oxidative stress, ↓ MDA content, ↑ SOD	[127]
	Lycopene	↓ NADPH oxidase, ↓ ROS production	[128]
	Lutein	↑ SOD, ↓ ROS level, ↑ CAT, ↑ GPx, ↓ GR, ↓ MDA, ↑ reduced glutathione level	[129]
	Zeaxanthin	↓ Myeloperoxidase, ↓ MDA, ↑ SOD, ↑ CAT, ↑ glutathione level	[130]
Polyphenols	Anthocyanins	Scavenged free radicals, ↑ SOD, ↑ total antioxidant activity	[120]
	Cyanidin-3-arabinoside	↓ Renal oxidative stress (↑ SOD, ↑ CAT), ↓ lipid peroxidation (↓ TBARS and ↓ MDA)	[121]
	Gy3G, Mv3G	↓ ROS, sustained the level of GSH and glutathione antioxidant defense system	[122]
	Petunidin-3,5-O-diglucoside	Scavenged free radicals, ↓ ROS, ↓ MDA level and GSH consumption	[123]
	Anthocyanin extract from purple highland barley	Scavenged free radicals, ↓ ROS, ↑ SOD, ↑ CAT	[131]
	Curcumin	↓ Serum MDA, ↑ total antioxidant activity, ↑ transcription and expression levels of antioxidant enzymes, ↑ mitochondrial function	[124,125]
Alkaloids	Betalain	↓ MDA, ↑ CAT, ↑ SOD, ↑ GPx, ↑ xanthine oxidase	[132]
	Betanin	Scavenged free radicals, ↓ MDA, ↑ total antioxidant activity	[133]

**Table 4:** The mechanism of some natural pigments for anti-cancer activity.

Cancers	Compounds Name	Category	Mechanism	Refs.
Breast cancer	Lycopene	Carotenoids	Activation of ERK1/2, ↓ cyclin D1 ↑ p21 ↓ phosphorylation of Akt and its downstream molecule mTOR ↑ Bax	[154]
	β-Carotene	Carotenoids	↑ Apoptosis ↓ cell cycle ↓ PI3K/Akt ↓ ERK	[155,156]
	Lutein	Carotenoids	↓ Breast cancer cell proliferation, ↑ expression of cellular antioxidant enzymes, ↓ ROS, ↑ NrF2/ARE pathway, ↓ NF-κB signaling pathway ↑ p53, ↓ HSP60	[157,158]
	Crocin	Carotenoids	↑ Disrupting the microtubule network ↓ Wnt/β-catenin target genes	[159,160]
	Astaxanthin	Carotenoids	↓ Cellular migration, ↓ cell number ↓ Expression levels of pontin, mutp53, Oct4, and Nanog, ↓ proliferation Activation of Bax/Bcl2, cleaved caspase-3, and cleaved caspase-9 as well as the phosphorylation of ERK1/2, JNK, and p38	[143,144,145]
	D-3-5-D, C3R	Polyphenols	↑ Intracellular reactive oxygen, ↑ apoptosis ↓ MCF-7 cells in the G2/M phases	[161]
	C3G, Pg-3-G	Polyphenols	↓ AMPK, ↑ apoptosis ↑ Oxidative stress	[162]
	Curcumin	Polyphenols	↓ NF-κB signaling pathway ↓ HER2-TK ↓ Akt protein, ↓ ubiquitin-proteasome pathway ↓ PI3K/Akt signaling pathway ↓ EGFR signaling	[163,164,165,166,167]
	Betanin	Alkaloids	↑ Apoptosis-related proteins (Bad, TRAILR4, FAS, p53)	[168]
Colorectal Cancer	Astaxanthin	Carotenoids	↓ Invadopodia, ↓ EMT, ↑ E-cadherin, ↓ vimentin, ↓ cortactin, ↓ MMP2, ↑ miR-29a-3p, ↓ ZEB1, ↓ MYC ↑ Apoptosis, ↑ Bax, ↑ caspase-3, ↓ Bcl2	[169,170]
	Fucoxanthin	Carotenoids	↓ Proliferation ↑ DNA damage	[171,172]
	Crocin	Carotenoids	↑ Caspase-3 and -7, ↓ proliferation	[173]
	C3G, C3XR, C3R	Polyphenols	↑ Probiotics, ↓ inflammation ↓ Pathogenic bacteria	[174]
	C3G, C3XR, C3R	Polyphenols	↑ MiR-24-1-5p, ↓ β-catenin	[175]
	Pg-3-G	Polyphenols	↓ HT-29 colon cancer cells	[176]
	Anthocyanin extract	Polyphenols	↓ Wnt/β-catenin ↓ Mitochondrion-mediated apoptosis	[146]
	Curcumin	Polyphenols	↓ NF-κB pathway, ↓ cell cycle ↑ Cytochrome c, ↑ Bax and p53, ↓ Bcl-2	[177,178]
	Betaxanthin and betacyanin	Alkaloids	↓ Bcl2-like protein 4, ↓ cleaved poly ADP-ribosyl polymerase 1, ↓ cleaved caspase-3 ↓ Anti-apoptotic protein B-cell leukemia/lymphoma 2 levels	[179]
Gastric cancer	Crocin	Carotenoids	↓ KLF5 HIF-1, ↑ miR-320, ↓ epithelial–mesenchymal transition, ↓ migration	[180]
	β-Carotene	Carotenoids	↓ Cell viability, ↑ DNA damage, ↑ apoptotic indices, ↑ caspase-3, ↓ Ku70/80	[181]
	Fucoxanthin	Carotenoids	↑ Beclin-1, ↑ LC3, ↑ cleaved caspase-3 (CC3), ↓ Bcl-2, ↓ cell cycle, ↑ apoptosis, ↓ Mcl-1, STAT3, and p-STAT3	[182,183]
	Astaxanthin	Carotenoids	↓ Cell cycle ↑ NADPH oxidase activity, ↑ ROS levels, ↑ LDH release, ↑ the number of propidium iodide-positive cells ↑ RIP1/RIP3/MLKL signaling pathway	[184,185]
	Curcumin	Polyphenols	↓ STAT3 pathway	[178]
Liver cancer	Astaxanthin	Carotenoids	↑ Cell number in G2 phase ↑ Cell number in G2/M phase ↑ Apoptosis ↑ Oxidative stress, ↑ adiponectin	[186,187,188]
	Crocin	Carotenoids	↓ NF-κB, ↓ inflammation, ↓ cell cycle, ↑ apoptosis	[189]
	Fucoxanthin	Carotenoids	↓ Glutathione (GSH) content, ↓ proliferation Reverting body weight, serum albumin, antioxidant enzymes, all the liver enzymes, serum bilirubin, and stress markers to normal levels in hepatocellular carcinoma rats	[190,191]
	C3G, Pn-3-G	Polyphenols	↓ TNF-α, iNOS, NF-κB ↓ Cell proliferation	[192]
	C3G, C3R	Polyphenols	↓ Lipid peroxidation, ↓ COX-2 ↑ Nrf2-mediated antioxidant enzymes	[193]
	M3G	Polyphenols	↓ Proliferation, ↑ apoptosis, ↓ ROS, ↑ JNK/p38 MAPK pathways, ↓ AKT phosphorylation, ↓ migration, ↓ invasion	[147]
	Curcumin	Polyphenols	↓ Migration, ↓ invasion, ↓ epithelial–mesenchymal transition, ↓ aryl hydrocarbon receptor/ERK/SK1/S1P3 signaling pathway	[194]
	Betanin	Alkaloids	↑ Nrf2, ↑ mitogen-activated protein kinases	[195]
Lung cancer	Astaxanthin	Carotenoids	↑ Cell number in G0/G1 phase ↑ p38 MAPK ↑ Apoptosis	[196]
	Crocin	Carotenoids	↑ G0/G1 arrest, ↑ mRNA levels of p53 and Bax, ↓ Bcl-2, ↑ apoptosis	[197]
	Lutein	Carotenoids	↓ PI3K/AKT, ↑ apoptosis	[198]
	C3G	Polyphenols	↓ Lung tumor multiplicity and tumor area, ↓ expression of proliferative cell nuclear antigen (PCNA) and Ki-67	[199]
	Curcumin	Polyphenols	↓ NF-κB, ↓ JAK2/STAT3 signaling pathway, ↓ JAK2 ↓ Cell proliferation, ↑ apoptosis ↑ microRNA-192-5p, ↓ PI3K/Akt signaling pathway ↓ Wnt/β-catenin pathway	[150,151,152,153]
	Betalain	Alkaloids	↑ Proliferation, ↓ cell cycles, ↑ p53/p21, ↓ levels of cyclin-D1 complex, ↓ levels of p-PI3K, ↓ p-Akt, ↓ mammalian target of rapamycin	[200]
Prostate cancer	Astaxanthin	Carotenoids	↑ Apoptosis, ↑ cleaved caspase-3; ↑ miR-375 and miR-487b	[201]
	Crocin	Carotenoids	↓ Proliferation, ↓ cell cycle, ↑ apoptosis ↓ Bcl-2, ↓ Bax	[202]
	Proanthocyanidins	Polyphenols	↓ Notch1 pathway	[203]
	C3G	Polyphenols	↓ Epithelial–mesenchymal transition	[204]
	Curcumin	Polyphenols	↓ Expression of CYP11A1 and HSD3B2, ↑ AKR1C2, ↓ dihydrotestos terone ↑ miR-34a, ↓ β-catenin, ↓ c-myc	[205,206]
